# Preliminary molecular characterization of foot-and-mouth disease virus SAT-1 topotype III associated with recent cattle outbreaks in Syria

**DOI:** 10.1371/journal.pone.0353862

**Published:** 2026-07-14

**Authors:** Morshed Kassouha, Yaseen Almohsen, Hazem Altaweel, Abdulkarim Hallak, Abdulkarim Kalballouz

**Affiliations:** 1 Department of Microbiology, Faculty of Veterinary Medicine, Hama University, Hama, Syria; 2 Department of Animal Diseases, Faculty of Veterinary Medicine, Hama University, Hama, Syria; 3 Department of Animal Diseases, Faculty of Veterinary Medicine, Idlib University, Idlib, Syria; 4 Department of Public Health and Preventive Medicine, Faculty of Veterinary Medicine, Hama University, Hama, Syria; Central Laboratory for Evaluation of Veterinary Biologics, Agricultural Research Center, EGYPT

## Abstract

Foot-and-mouth disease (FMD) is a highly contagious transboundary viral infection that poses a significant threat to the livestock industry globally. In Syria, despite ongoing vaccination programs, recent outbreaks (2025–2026) in cattle and sheep has raised concerns due to insufficient antigenic coverage and rapid transmission. This study aimed to identify and molecularly characterize the FMD virus (FMDV) responsible for the disease in six cattle farms across Hama and Homs governorates in January 2026. Samples from teat vesicular fluid and oral swabs were collected and analyzed using RT-PCR targeting the VP1 region. The six farms tested positive for the SAT-1 serotype, and two positive isolates: Syr-Hama 2026 and Syr-Homs 2026, were sequenced and subjected to phylogenetic analysis using the WRLFMD genotyping tool and MEGA11 software. Molecular analysis identified the causative agent as FMDV serotype SAT-1, marking its first molecularly confirmed detection and molecular characterization of this serotype in Syria. Further genotyping revealed that both isolates belong to Topotype III. The Syrian isolates exhibited high nucleotide identity (98.9%) with each other and significant similarity to sequences recently recorded in Lebanon, Azerbaijan, Iran, and Turkey. Phylogenetic tree confirmed a close relationship with the reference strains such as BOT/1/77. The emergence of SAT-1/III in Syria, alongside the co-circulation of the O/ME-SA/SA-2018 lineage, leads us to hypothesize a complex epidemiological shift likely driven by unregulated animal movement across borders. Although this study has its limitations and is based on a limited sample size, the results underscore an immediate necessity to assess and, if required, revise national vaccination strategies, as well as expanded nationwide surveillance and antigenic characterization are required to determine the diversity of SAT-1 strains circulating in Syria and to inform evidence-based vaccine strain selection.

## 1. Introduction

Foot and Mouth Disease (FMD) is recognized as one of the most perilous transboundary viral infections impacting cloven-hoofed animals, such as cattle, buffalo, sheep, goats, and pigs, along with certain wild species. The disease is noted for its swift transmission through various channels, such as the movement of animals and humans, contaminated animal products (including meat, milk, and semen), equipment and clothing, and even via airborne particles. This characteristic renders it a significant factor in international trade limitations and substantial economic detriment to the livestock industry. [1–3] The pathogen responsible for this disease is the foot-and-mouth disease virus (FMDV), which is a non-enveloped, single-stranded RNA virus (+ssRNA) classified under the genus Aphthovirus within the family Picornaviridae. The genome of FMDV is roughly 8.5 kilobases in length and is encased in an icosahedral capsid formed by four structural proteins: VP1, VP2, VP3, and VP4. Among these, VP1 is the most antigenically significant protein, and its genetic sequence is utilized for virus classification and for elucidating genetic relationships among various isolates. [4,5] There exist seven distinct serotypes of the virus (O, A, C, Asia-1, SAT1, SAT2, and SAT3), which do not confer cross-immunity. Each serotype comprises numerous subtypes due to a high rate of mutation. The geographical distribution of these serotypes is not uniform. For instance, serotypes O and A are predominantly found in Africa and Asia, whereas SAT serotypes have historically been restricted to sub-Saharan Africa, although recent outbreaks have been documented in other continents and regions. The Asia-1 serotype is present in Central and South Asia, with no cases of serotype C reported since 2004. [6–8].

Infected animals display clinical signs, particularly characterized by bullous lesions in the mouth, feet, and udder teats, along with symptoms such as fever, reduced appetite, and diminished milk yield. Young animals experience high mortality rates due to cardiac complications. Certain wild species, including the African buffalo, may act as a persistent reservoir for the virus, facilitating periodic transmission to livestock. [1,4] A variety of diagnostic techniques have been established, encompassing traditional methods like virus isolation and competitive and antigen-blocking ELISAs, as well as molecular techniques such as RT-PCR and RT-LAMP. Although different ELISA methods provide commendable diagnostic sensitivity and specificity, molecular detection techniques offer the benefit of superior diagnostic and analytical sensitivity for identifying trace amounts of viral RNA [8]. In the last twenty years, molecular methodologies such as PCR and DNA nucleotide sequencing have greatly improved our comprehension of disease epidemiology by revealing the geographic topotypes, subtypes, and genotypes of circulating viruses. This advancement has enabled global surveillance of viral dissemination and the formulation of more accurate control measures. Despite the implementation of vaccination initiatives in numerous countries, the absence of cross-protection among serotypes and the variability of strains results in persistent outbreaks, as recently observed in Egypt, Germany, Slovakia, Hungary, and Bangladesh. This highlights the critical necessity for ongoing monitoring, updating, and production of vaccines specifically designed for locally prevalent serotypes. [9–11].

Historically, there has been a lack of information regarding the circulation of foot-and-mouth disease virus (FMDV) in Syria. The limited samples collected from Syria for serotype characterization were obtained during the 1960s, 1980s, and 1990s, and subsequently sent to the World Reference Laboratory for Foot-and-Mouth Disease (9 samples). The identified serotype was type O. Additionally, some samples were forwarded to the same laboratory in 2002 (4 samples), revealing the presence of the viral lines O/ME-SA/PanAsia and A/ASIA/Iran-96. This information was sourced from the GenBank database and the www.fmdbase.org database. Between 2002 and 2025, there were no studies, published research, or references to a specific sequence of this virus originating from Syria.

In 2025, however, a serological study was published that took place in northwestern Syria [12], aimed at assessing immunity following a vaccination campaign in those regions, which confirmed the importance of the O serotype. Recently, at the start of 2026, eight sequences were deposited in the Gene Bank and the www.fmdbase.org database. These sequences were collected from northern and eastern Syria and were also identified as the O serotype. All the aforementioned factors regarding the scarcity of information, the inadequacy of epidemiological surveillance of the disease, the lack of expertise and resources, the instability in the region, and the deficiencies of veterinary health authorities in certain countries have contributed to the conditions and environment that have led to outbreaks affecting Syria in both the past and the current year.

In light of the recent outbreaks of foot-and-mouth disease virus in cattle across various Syrian governorates during 2025 and 2026, which have been paralleled by similar incidents in neighboring nations, particularly Lebanon, it was crucial to ascertain the serotype, topotype, and subtype for any forthcoming strategic initiatives focused on the production or importation of vaccines to manage the disease. Consequently, this study was conducted to identify the foot-and-mouth disease virus in suspected cattle cases, to characterize it at a molecular level, to analyze its correlation with the viruses prevalent in adjacent countries, and to propose necessary measures for disease control in Syria.

## 2. Materials and methods

### 2.1. Study areas, Samples collection and preservation

Outbreaks of foot-and-mouth disease (FMD) among cattle and sheep have been reported across most governorates of Syria. These outbreaks commenced around the middle of 2025 and persist to the present during 2026. Additionally, outbreaks have been noted in adjacent countries, particularly Lebanon.

**Fig 1:**
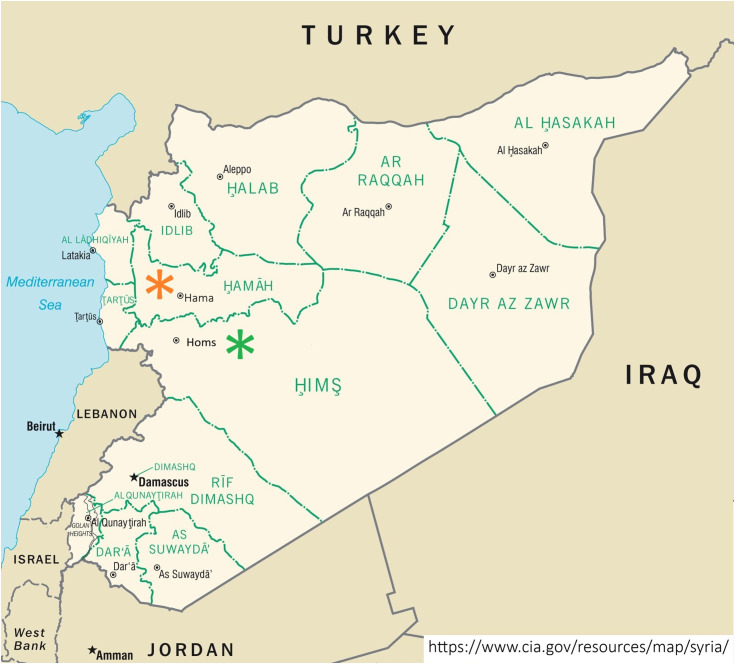
Map of Syria and the surrounding countries. The two governorates from which the samples were collected: Hama (orange asterisk) and Homs (green asterisk). Adapted from a base map by the Central Intelligence Agency (CIA), public domain. This figure is similar but not identical to the original image and is therefore for illustrative purposes only.

For the purpose of this study, six cattle farms located in the rural regions of the Homs and Hama governorates were examined in January 2026 (Fig 1), all of which had documented instances of FMD (four farms in Hama and two in Homs).

**Fig 2:**
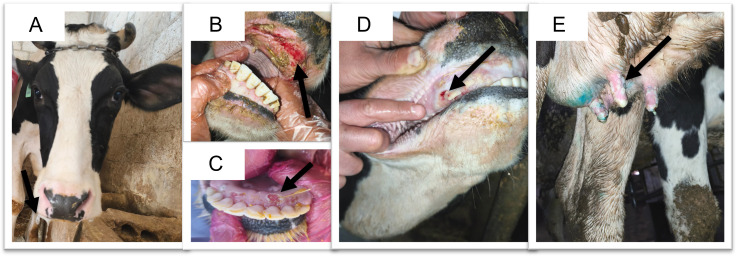
Clinical observations and lesions in cattle infected with FMDV in a natural setting. (A) Excessive salivation observed in infected cattle. (B) Recently ruptured hemorrhagic vesicles. (C, D) Formation of ulcers on the dental pad. (E) Presence of vesicles on the teats of the udder.

The typical symptoms of the disease were evident, particularly the presence of vesicles in the mouth, on the muzzle, tongue, teats, and hooves (Fig 2).

Following the acquisition of verbal consent from the farmers for the collection and utilization of samples for research objectives, samples were collected from the fluids of the vesicles located on the teats of the udders, as well as swabs from newly ruptured and hemorrhagic vesicles in the mouth of the affected animals.

It is crucial to highlight that before gathering animal samples, authorization was secured from the Hama University Ethics Committee (514–11/2026), which was contingent upon the endorsement from the Scientific Research and Animal Welfare Ethics Committee at the Faculty of Veterinary Medicine (95–2025). The research team collected all samples without the use of anesthesia, prioritizing the comfort of the animals and complying with all required sterilization protocols, especially when utilizing needles to extract fluid from the vesicles located on the teats, and we confirm that no animal was euthanized.

These samples were placed in plastic tubes containing a 0.04M phosphate buffer, with glycerol making up 50% of the solution [13], and the relevant data for each sample was documented on the tube. The samples were then stored in an icebox and transported to the Faculty of Veterinary Medicine at the Hama University, where they were preserved at −80˚C until further analyses were conducted.

### 2.2. RNA extraction and cDNA synthesis

Viral RNA was extracted from the phosphate buffer following adequate vortexing, utilizing the **GF-1 Viral Nucleic Acid Extraction Kit (Vivantis, Malaysia)** in accordance with the manufacturer’s guidelines.

Subsequently, cDNA was synthesized employing **RevertAid First Strand cDNA Synthesis Kit (Thermo Scientific, Lithuania)** with random hexamer, adhering to the manufacturer’s protocols, incorporating 10 µL of the extracted RNA into the reverse transcription reaction.

The VP1 sequence was amplified via PCR using primers that are specific to serotypes O, A, and SAT-1 (Table 1) [7], which are deemed the most probable based on prior studies and some unpublished data from adjacent countries. In the event of a negative result, primers for the other four serotypes were utilized, which were previously obtained with the three previous serotype primers from **(Macrogen, Inc., Korea)**.

**Table 1:** Sequence, virus serotypes specificity, and size of PCR amplification product of oligonucleotide primers.

No.	Oligo Name	5` - Oligo Seq – 3`	Product size bp	Used for
1	O–1C272F	TBGCRGGNCTYGCCCAGTACTAC	1135	O Serotype
2	O–1C244F	GCAGCAAAACACATGTCAAACACCTT	1165	O Serotype
3	O–1C283F	GCCCAGTACTACACACAGTACAG	1124	O Serotype
4	A–1C562F	TACCAAATTACACACGGGAA	866	A Serotype
5	A–1C612F	TAGCGCCGGCAAAGACTTTGA	814	A Serotype
6	EUR–2B52R	GACATGTCCTCCTGCATCTGGTTGAT	-----	Serotype O/ A
7	SAT1–1C559F	GTGTATCAGATCACAGACACACA	1043	SAT-1 Serotype
8	SAT1U–OSF	GTGTACCAGATCACTGACAC	1043	SAT-1 Serotype
9	SAT1–P1–1228F	AACCTGCACTTCATGTACAC	1286	SAT-1 Serotype
10	SAT–2B208R	ACAGCGGCCATGCACGACAG	----	All SAT serotypes

### 2.3. PCR analysis

The synthesized cDNA was used as a template for PCR amplification of VP1 of the FMD virus genome using specific primers for each FMDV serotype.

The PCR amplification was performed in a 50 μL volume containing 4 μL cDNA, 1 μL dNTPs (10 mM), 1 μL of each primer (10 μmol), 1.5 μL MgCl_2_ (50 mM),5 μL (10×) Taq buffer, 0.5 μL Taq polymerase (Vivantis, Malaysia), and 36 μL nuclease-free water. The PCR thermal cycling protocol commenced at 94˚C for a duration of 5 minutes, constituting one cycle; this was succeeded by 35 cycles of: 94˚C for 1 minute. The annealing temperature was determined by the specific target serotype, with settings of 50˚C for the SAT-1 serotype, 55˚C for the A serotype, and 60˚C for the O serotype, each held for 1 minute. [7], The extension phase occurred at 72˚C for 2 minutes; ultimately, a final extension cycle was performed at 72˚C for 5 minutes. All PCR reactions were carried out using an Techne thermal cycler (Tc-512). nuclease-free water was used as a negative control. The PCR products were visualized by electrophoresis on a 1.5% agarose gel stained with ethidium bromide.

DNA products were gel-purified using a **PCR Clean-Up & Gel Extraction Kit (GeneDirex, China)**, according to the manufacturer’s instructions, and the concentration of the purified DNA was then determined using a Nanodrop instrument (Thermo). The purified DNA was stored at −20 °C until sequencing.

### 2.4. Sequencing and sequence analysis

The six farms tested positive for the SAT-1 serotype, and PCR products of two samples, one sourced from Homs Governorate and the other from Hama Governorate, were purified and sequenced utilizing sanger platform of the Macrogen Sequencing System **(Macrogen, Inc., Korea)**. The recommended primers for the sequencing were adopted according to the Pirbright Reference Laboratory protocol scheme [7].

Multiple PCR products were sequenced for each sample in both directions and were matched to confirm the validity of the results; no sequence assembly was required to obtain the complete VP1 fragment.

The sequences chromatograms were manually inspected with the aid of Geneious Sequencing Software version 4.8.4., ensuring the quality and accuracy of the results and the signal, and then aligned. Subsequently, the resulting sequences underwent analysis through the FMDV Genotyping Tool (FAO World Reference Laboratory for Foot-and-Mouth Disease, WRLFMD) and BLAST analysis via the NCBI GenBank BLAST tool (Basic Local Alignment Search Tools, http://www.ncbi.nlm.nih.gov/blast). Both sequences were submitted to the NCBI GenBank database. Nucleotide sequences corresponding to reference serotypes and topotypes were retrieved from the GenBank database and integrated with our sequences to facilitate the construction of a phylogenetic tree.

The GenBank database was additionally examined for any sequences submitted in recent years from Syria, even though we are cognizant of all research conducted by universities and governmental organizations focused on animal diseases.

### 2.5. Phylogenetic analysis

The phylogenetic tree was constructed with Mega11 software by using the maximum likelihood (ML) method based on the Tamura-Nei model, 1000 bootstrap replicates. This phylogenic analysis included sequences of the two Syrian sequences from this work, and 33 sequences of SAT-1 topotypes obtained from the GenBank.

## 3. Results

### 3.1. Detection and classification of FMDV serotypes

A uniform approach was implemented for all samples obtained from the six farms. RNA was extracted from two samples from each cattle farm and subsequently analyzed using RT-PCR with all the primers detailed in (Table 1) for each serotype. This methodology aimed to enhance the likelihood of virus detection and to amplify the VP1 protein sequence, irrespective of whether the serotype identified was A, O, or SAT-1.

The findings indicated the presence of DNA bands in the reactions specific to the SAT-1 serotype across samples from all cattle farms that displayed symptoms of foot-and-mouth disease. No positive results were observed for any of the other serotypes. This finding marks the first molecularly confirmed detection of the SAT-1 serotype in Syria.

### 3.2. VP1 region sequencing and sequence analysis

Following the PCR results indicating that the serotype accountable for the foot-and-mouth disease virus outbreak in the cattle farms examined in this study was SAT-1, two samples were chosen based on having the highest concentration of the PCR product. One sample was sourced from Homs Governorate **(Syr-Homs 2026)**, while the other was from Hama Governorate **(Syr-Hama 2026)**, and both were dispatched for sequencing analysis at **Macrogen**. To ensure sequence fidelity, replicates of each isolate were sequenced in both forward and reverse directions.

Following the acquisition of the sequence results and the identification of the VP1 sequence for both samples, a BLAST analysis was conducted alongside the WRLFMD genotyping tool. The findings from both analyses were consistent, revealing that the two viruses present in the cattle farms are classified under the same topotype, specifically Topotype III.

Upon aligning the nucleotide sequences of VP1 from the two isolates, a difference was observed in the positions of seven nucleotides out of a total of 663 for the entire fragment, indicating an identity rate of 98.9%. Nevertheless, following the alignment of the amino acids derived from these sequences, it was noted that six of the differences were silent mutations, which did not result in any alteration of the amino acids, with the exception of a single nonsynonymous mutation at the seventh amino acid position, where glycine in Syr-Hama 2026 was substituted by serine in Syr-Homs 2026.

Accession numbers were acquired for the sequences of the two isolates from the gene bank (NCBI), specifically PZ124873 and PZ124874, corresponding to the Hama and Homs isolates, respectively.

### 3.3. Phylogenetic analysis

Following the identification of the serotype and topotype of the foot-and-mouth disease virus, the subsequent step involved examining the similarity and identity of the VP1 sequence from the Syrian isolates with those of foot-and-mouth disease viruses from various SAT-1 topotypes, in addition to established reference sequences and viruses documented in neighboring countries adjacent to Syria. This analysis aimed to develop a hypothesis or preliminary evidence regarding the origin of the virus responsible for the outbreak.

**Fig 3:**
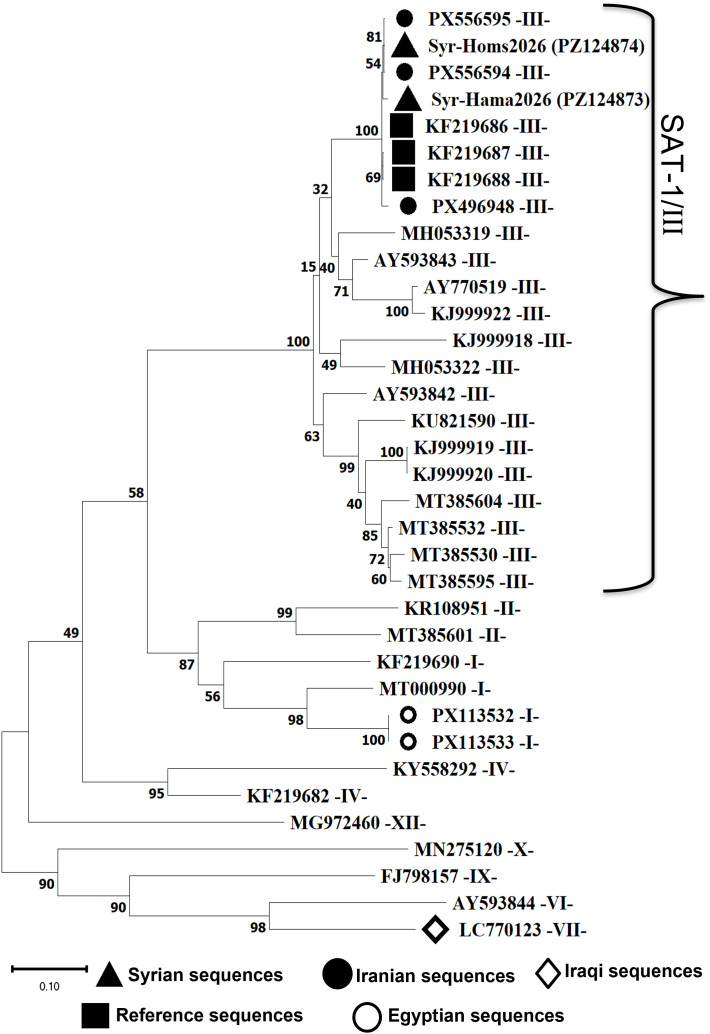
Maximum Likelihood phylogenic tree using Tamura-Nei model (1000 bootstrap replication), showing the relationship between the VP1 sequences of the Syrian Isolates with other sequences of serotype SAT-1 topotypes, available in the GenBank database and prototype strains (reference sequences) of topotype III.

**Fig 4:**
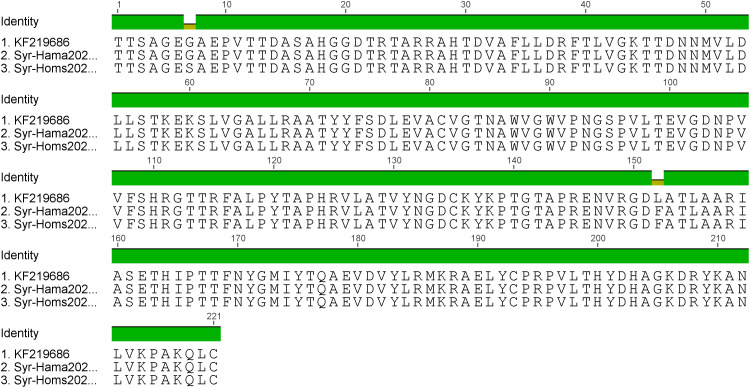
A comparative analysis of the amino acid sequences of the VP1 protein derived from the two Syrian isolates alongside the reference strain BOT/1/77 (KF219686), which reveals the only two sites of divergence that can be observed in the green discontinuities.

Upon conducting a study of the sequences through BLAST analysis, the VP1 sequences of the SAT-1 serotype were acquired, assessed, and a phylogenetic tree was generated utilizing Mega11 software (Fig 3). A notable similarity was identified between the Syr-Homs2026 sequence and several analogous viral sequences sourced from Iran [14], with identity rate ranged between 99.7 and 99.39. It is crucial to highlight that the sequences from the Syrian isolates exhibited a high degree of similarity to significant reference sequences, including the BOT/1/77 strain sequence (accession number KF219686). The identity rate of this reference strain with the Syr-Hama 2026 sequence was recorded at 99.1%, while with the Syr-Homs 2026 sequence, it was 99.5%. However, an analysis of this reference sequence in terms of amino acids revealed a difference of only one amino acid at position 152 when compared to the Syr-Hama 2026 sequence, and a difference of two positions when compared to the Syr-Homs 2026 sequence: one at position 152 and another at position 7 (Fig 4).

Utilizing the WRLFMD genotyping tool, the VP1 protein sequence derived from Syrian isolates exhibited a significant level of similarity to the SAT-1/III virus sequences. These sequences were collected during the outbreaks of 2025; however, they have not yet been published, which limits our ability to provide additional information regarding them. Nonetheless, it is important to highlight that a review of the most recent quarterly report for 2025 https://www.wrlfmd.org/sites/world/files/quick_media/WOAH-FAO%20FMD%20Ref%20Lab%20Report%20Oct-Dec%202025%20v2.pdf, released by the European Commission for the Control of Foot-and-Mouth Disease, FAO, and the Foot-and-Mouth Disease Reference Laboratory, revealed that virus sequences collected from Turkey, which shares a border with Syria, and Iran in November [14] were highly identical to those of the reference strain (BOT/1/77). Consequently, it can be inferred that they align closely with the sequences obtained from the outbreaks in Syria. Furthermore, the same report indicated that samples from Lebanon, another neighboring country to Syria, and Azerbaijan were classified as belonging to the SAT-1/III type, suggesting a likelihood of similarity to the sequences found in Syria [15].

The constructed phylogenetic tree (Fig 3) corroborated all aforementioned findings. Additionally, sequences documented in 2025 from Egypt (accession numbers PX113532, PX113533), and in 2024 from Iraq (accession number LC770123) were found to be significantly divergent from the Syrian sequences, despite belonging to the SAT-1 serotype, as they corresponded to topotypes I and VII, respectively.

A final significant discovery during our investigation, according to the GenBank database, reveals that 8 new Syrian sequences have released at the onset of 2026 for samples collected from Deir ez-Zor Governorate and Hama Governorate in 2025(accession numbers PX685507–14). These samples were sequenced and genotyped in Turkey by Turkish researchers. All samples were classified as the O/ME-SA/SA-2018 lineage, exhibiting a very high identity rate among them, with the variation in amino acids within the VP1 sequence not exceeding one amino acid.

## 4. Discussion

Syria is categorized under POOL 3, as per the classifications established by the FAO, its World Reference Laboratory for Foot-and-Mouth Disease (WRLFMD), and the World Organization for Animal Health (WOAH). This region includes countries in West Asia, the Gulf States, and the Middle East [12]. The significance of this area is heightened by its political instability, ongoing conflicts, and overall precarious conditions. Such instability undermines the effectiveness of national measures and regulations essential for controlling, limiting, and preventing the spread of foot-and-mouth disease viruses, particularly between POOL 2 and POOL 4 on one side, and POOL 3 on the other. This phenomenon was evident between 2023 and 2025 with the emergence of the O/ME-SA/SA-2018 strain in Iraq and Bahrain (which originated from POOL 2 countries), alongside the SAT-1/I and SAT-2/XIV strains [16,17]. For 14 years, Syria has been engulfed in chaos and conflict, leading to a deterioration in the health services available for livestock and a lack of effective border control, which has facilitated the illegal and unregulated movement of people, animals, and their products from neighboring nations. The ability of governmental and non-governmental organizations to secure and distribute vaccines to animals in conflict-affected areas has also been compromised. Given that specific serotypes O, A, and Asia-1 are recognized as endemic in the POOL 3 region, the vaccines utilized in Syria for many years have included strains of these serotypes [12]. These strains were identified based on reports from the WRLFMD reference laboratory and data from adjacent countries, without any actual laboratory analysis based on samples collected within Syria, as the last samples documented before 2025 by the www.fmdbase.org database and the GenBank date back to 2002 (4 samples in which the viral lines O/ME-SA/PanAsia and A/ASIA/Iran-96 were found).

For the last twenty years, a vaccine has been officially imported via the Ministry of Agriculture from a sole source to manage foot-and-mouth disease in Syria. Until 2023, this imported vaccine included the A/Iran-5, O/PanAsia 2, and Asia-1 strains [href:https://rr-middleeast.woah.org/app/uploads/2022/09/7-syria-2022_gf-tads_fmd-ppr_template.pdf]https://rr-middleeast.woah.org/app/uploads/2022/09/7-syria-2022_gf-tads_fmd-ppr_template.pdf. Following 2023, the SAT-2 Eritrea 98 strain was incorporated at the request of the veterinary health authorities, prompted by reports of its dissemination in several neighboring countries, especially Iraq [16,18], despite the absence of any laboratory scientific evidence confirming its existence in Syria. Concurrently, FAO supported separate vaccination campaigns in northwestern Syria(the Idlib and Aleppo governorates) using a formulation that contained the A Nep 84, A Iran-5, O PanAsia-2, and Asia-1 Tur 15 strains [12].

This study represents the first of its kind conducted in Syria, aiming to genotyping the foot-and-mouth disease virus from the outbreaks and clinical cases to gain insights into the actual situation on the ground.

In over 40 years, only one scientific paper has been published on the reality of foot-and-mouth disease in Syria, in 2025. This paper was a serological study conducted in some areas of northern Syria (Aleppo and Idlib governorates). This study aimed to assess antibody levels following a vaccination campaign supported by the FAO during 2021 and 2022 in response to the increasing cases of FMD in those areas. The study also investigated the presence of antibodies to non-structural proteins to determine the presence of natural field infection. The findings indicated the importance of serotype O and suggested it was the most prevalent serotype affecting animals in those regions [12].

More recently, around the middle of 2025, a new outbreak of foot-and-mouth disease emerged in Syria, affecting cattle and sheep herds. This outbreak was notable for its high contagious character and insufficient antigenic coverage of the existing vaccines to provide adequate protection, despite Syria’s classification as a nation that manages the disease through vaccination efforts.

This research was initiated in response to the outbreak and its extension to the Homs and Hama governorates, which are in proximity to the Faculty of Veterinary Medicine. Samples were gathered from six cattle farms within these two governorates, and RT-PCR testing molecularly confirmed that serotype SAT-1 was the responsible agent for this outbreak. Following the sequencing and analysis of two isolates, one from Homs and another from Hama, it was determined that the VP1 serotype belonged to Topotype III.

Instances of foot-and-mouth disease outbreaks were reported in Syria and Lebanon concurrently, and this research, in conjunction with the report of EUFMD, FAO, WRLFMD, [15] suggested that the viruses responsible for these outbreaks in both nations were of the SAT-1/III type. It is hypothesized that the outbreaks in both nations stemmed from a common source, considering the rampant illegal and uncontrolled smuggling of animals across borders, along with the movement of individuals. Nevertheless, determining which country first experienced the outbreak requires further investigation.

We also hypothesize that Egypt and Jordan are not the source of this virus (as some press reports have suggested), due to the absence of any similar viruses identified in scientific papers, gene bank sequences, or even the WRLFMD database (www.fmdbase.org) in recent years, up to the time of composing this article. All documented viruses have been of other serotypes [19] or of the SAT-1 serotype but with a different topotype, particularly SAT-1/I in Egypt [20]. On the other hand, the sequences of the Syrian foot-and-mouth disease viruses were largely identical to the viruses recently recorded in Iran and Turkey in 2025 (as explained in the Results section), as well as in Azerbaijan (SAT-1/III). This data supports the suggestion that the outbreak in Syria has an epidemiological linkage with the viruses from those regions, which may have reached Syria via Turkey or Iraq, even though scientific articles from Iraq did not mention the presence of SAT-1/III, nor were there any deposited sequences of this topotype, while there is evidence of the presence of the SAT-1/I and SAT-1/VII strains [15,17]. It is important to highlight those earlier assumptions regarding the origin and transmission pathway of the virus responsible for the outbreaks in Syria are purely speculative and necessitate further tracking, investigation, and validation to be established as facts.

The report from EUFMD, FAO, WRLFMD, [15] highlights a critical and significant hypothesis: veterinary authorities in the countries where these outbreaks first occurred must verify that the virus did not escape from vaccine production facilities into the field, nor from a vaccine that was not fully inactivated. This verification is especially crucial given the considerable similarity between the virus causing these outbreaks in all nations and the known strain used in vaccines (BOT/1/77). This significant and serious matter was also referenced in the recently published article concerning the same SAT-1/III strains that led to the recent outbreaks in Iran [14].

It is crucial to highlight that in 2025, eight clinical samples of foot-and-mouth disease were gathered from the governorates of Deir ez-Zor and Hama, and subsequently genetically sequenced by Turkish researchers in Turkey. All of these samples were classified under the O/ME-SA/SA-2018 lineage, which several reports indicate originated in POOL 2 and has spread in recent years to countries in POOL 3, including Syria, this suggests a complicated epidemiological scenario for this disease in Syria. This situation calls for continuous research and the establishment of a local reference laboratory within Syria to examine this virus and other transboundary diseases, thereby providing international organizations with the most recent updates and real-time epidemiological information.

While the detection of SAT-1/III highlights a potential gap in current vaccination strategies, expanded nationwide surveillance and antigenic characterization are required to determine the diversity of SAT-1 strains circulating in Syria and to inform evidence-based vaccine strain selection [21,22]. Ongoing studies should be pursued and finalized to explore the circulating strains throughout Syria.

Ultimately, it is crucial to acknowledge the constraints of this research, particularly the limited number of samples that were analyzed and sequenced. This investigation serves as an initial exploration, laying the groundwork for subsequent, more extensive and detailed studies.
