# Supplementary material for: Preliminary molecular characterization of foot-and-mouth disease virus SAT-1 topotype III associated with recent cattle outbreaks in Syria
Source: PLoS One. 2026 Jul 14;21(7):e0353862. doi: 10.1371/journal.pone.0353862 (PMC13367722; doi:10.1371/journal.pone.0353862)
Supplement: Attachment — Submitted filename: Response to Reviewers.docx [file pone.0353862.s002.docx]

**Response Letter**

Dear Editor-in-Chief,

We would like to thank you and the Reviewers for your useful comments and revises about our manuscript. The detailed corrections are listed below:

| **Author's answer** | **Journal Requirements** |
| --- | --- |
| Done. | 1.Please ensure that your manuscript meets PLOS ONE's style requirements, including those for file naming. |
| Done. | 2. Please provide a complete Data Availability Statement in the submission form |
| Done, we replaced the figure 1 with one from another website ([Syria - CIA](https://www.cia.gov/resources/map/syria/)), and according to the source, we do not need their permission to use or modify it. | 3. We note that Figure 1 in your submission contain map images which may be copyrighted. |
| Figure 2 is composed of photographs captured by the authors, which have not been published previously, illustrating instances of foot-and-mouth disease from the farms where the samples were collected. We relinquish all rights to their usage to the journal and authorize their use by a third party or republication as considered suitable. | 4. We note that Figure 2 in your submission contain copyrighted images. |

| **Reviewer #1** | **Author's answer** |
| --- | --- |
| 1- Data Availability Statement is incorrect, the authors state: “No data was generated by this study.” This is factually incorrect. Two novel VP1 sequences were generated and submitted to GenBank (accessions PZ124873, PZ124874). The data availability statement must be revised accordingly, e.g.:“The VP1 sequence data generated during this study are available in GenBank under accessions PZ124873 and PZ124874. Other relevant data are within the manuscript.” | We completely agree.  Done |
| 2. Sample size and representativeness are very limited. Only six farms were sampled, with sequencing performed on just two isolates. Given the claim of a“complex epidemiological shift,”the authors should acknowledge this as a preliminary observation and avoid overgeneralizing to all of Syria. | Certainly, the number of samples is small and limited, and we fully agree with the reviewer's observation, which is why we wrote the word "preliminary" at the beginning of the title. On the other hand, the outbreaks that occurred in Syria during 2025 and the current year were extremely severe and their spread was terrifying, with most of the previously used vaccines proving ineffective. If we take this into account, along with the large spread that accompanied the outbreaks that occurred in Lebanon, the country neighboring Syria, which were also caused by FMDV SAT-1/III, this aligns with the result of this work, which definitively confirmed its presence in Syria for the first time. Furthermore, the presence of a new strain of the virus, serotype O(O/ME-SA/SA-2018 lineage), introduced into Pool-3 from Pool-2, as we mentioned in the discussion, was also recorded. All of the above points suggest a complex epidemiological situation that requires close monitoring, especially given the authorities' inability to control borders and animal movement, and the indiscriminate use of vaccines from various sources without any controls. |
| 3. Missing critical methodological details. PCR annealing temperatures are vaguely reported as “50, 55, or 60°C depending on serotype.”The exact temperature used for SAT-1 must be stated. | Done. (page 11) |
| 4. Phylogenetic tree presentation is poor.(1) Figure 3 is unreadable in the current format (font too small, branches unclear).(2) Bootstrap values are mentioned but not clearly shown on key nodes.(3) The tree should be simplified or split into a main figure + supplementary. | The figure has been modified to become clearer, and the reviewer can download the figure in full resolution from the link at the top of the page. Thank you for your note |
| 5. Unsupported claims in Discussion.(1) “Virus did not escape from vaccine production facilities”–no data support this. Speculation should be removed or clearly labeled as hypothesis.(2)“Egypt and Jordan are not the source” while plausible, absence of evidence is not evidence of absence. Tone should be more cautious. | Claim (1): We fully agree with the reviewer's statement, and it was emphasized that this is a hypothesis (page 21, line 4). It should be noted that what was mentioned in the discussion chapter was taken literally from the referenced WOAH/FAO/WRLFMD report, as well as from a recently published article that highlighted the significant similarity between the SAT-1/III foot-and-mouth disease virus, which caused large outbreaks in Iran, and the BOT/1/77 reference strain used in vaccine production. However, this claim certainly remains a hypothesis.  Claim (2): That's a correct point. As we hypothesized in the discussion section regarding this issue, we based our assumption on the evidence available in published scientific articles and the sequences deposited in the GenBank database or the International Reference Laboratory for Foot-and-Mouth Disease. It's worth noting that the veterinary authorities in Egypt and Jordan are among the best in the region, and both countries have universities with advanced laboratories capable of detecting the viruses causing outbreaks. |
| 6. Ethics statement issues. Verbal consent from farmers is noted but not in line with standard ethical guidelines for animal studies. Written consent or a formal waiver should be documented. The statement should clarify whether any animals were euthanized (apparently none). | Obtaining written consent is certainly preferable (especially for studies conducted on humans, regardless of their form).  In this study, it was impossible to obtain written consent from some farmers whose animals were sampled because they are illiterate. We explained to them that these samples collected from their animals would contribute to identifying the pathogen affecting their livestock, and that the results would be used and published in scientific research to enhance our understanding of this virus. They were happy to agree.  A statement was added indicating that no animals were euthanized in this study. (page 9, line 2) |
| 7.Writing & Language (1) Line 2 (page 7): “outbreak”→should be “outbreaks” (plural). Line 5 (page 7): “have raised”→“has raised” (referring to “recent outbreak”). Several sentences are overly long and complex; consider splitting for clarity. | Done. |
| 8. Results section. The finding of 8 Syrian O/ME-SA/SA-2018 sequences (page 20) is interesting but feels disconnected from the main SAT-1 focus. This should be briefly integrated into the Discussion, not just reported. | This point was reported at the end of the results, and the significance of the O/ME-SA/SA-2018 lineage is discussed in several places in the discussion section (page 22). |
| (1)Figure 2 (clinical photos) is useful but lacks scale bars or annotations. | The figure's annotations are detailed in the caption on page 9. However, given that the images depict clinical symptoms—some closely focused on the mouth to illustrate the lesions, while others are taken from a distance to capture the animal's full head and excessive salivation—it seems quite challenging to define a single scale bar that accurately represents the entire figure. |
| (2) Figure 4 (amino acid alignment) could be merged into a supplementary table. | We appreciate the reviewer's observation. While we are not rigidly attached to our viewpoint and are receptive to modifications, we propose keeping the figure with an added explanatory sentence in the caption. Figure 4 illustrates the variations in amino acids and their respective positions among the Syrian sequences, as well as in comparison to the significant reference strain BOT/1/77.  The reader can readily identify the two points of divergence where the green line is interrupted. The first point of divergence occurs between the sequence of the virus studied from Homs Governorate, Syria, and the other two sequences. The second point highlights the differences between the reference strain BOT/1/77 and the Syrian sequences. The green line aids in simplifying and enhancing the visualization of these differences, and we have included a sentence in the caption to clarify this. |

| **Author's answer** | **Reviewer#2** |
| --- | --- |
| No research on FMD virus for molecular characterization has been undertaken by Syrian scholars within Syria over the last forty years. All existing data regarding serotypes and the molecular characterization of the foot-and-mouth disease virus has been derived from samples collected by researchers outside of Syria, which were molecularly characterized at the WRLFMD laboratory during the 1960s, 1980s, 1990s, and in 2002. Furthermore, the sequences referenced in our article were molecularly characterized in Turkey in 2025 and subsequently deposited in GenBank at the start of 2026 (it is important to note that all serotypes mentioned were of types O and A). This situation arose because the state historically prohibited any research on foot-and-mouth disease, whether serological or molecular, due to its repercussions on the economy and animal exports. However, since 2025, such research has become feasible. Consequently, this study aligns precisely with the reviewer's comment, as it represents the first identification of the causative agent SAT-1 topotype III and its initial molecular characterization.  We included a concise historical overview in the introduction regarding the limited knowledge about Syria, and we will address all the requests made by the reviewer. (pages 6,2,21) | 1) Inadequate historical context of FMD in Syria (critical issue)  The manuscript does not provide a comprehensive epidemiological history of FMD in Syria and instead frames the findings as:  “first documented detection of SAT-1 in Syria”  This claim is insufficiently supported and potentially misleading, given that:  * FMD has been historically reported in Syria with multiple serotypes  * SAT-1 circulation has been documented historically in the region  Required revision  * Clarify whether this represents:  * first recent detection  * first molecular characterization  * or first identification of Topotype III  * Include a structured historical overview of FMD circulation in Syria |
| All vaccines that have been officially administered in Syria over the last twenty years, up to 2025, were imported from a singular supplier and were entirely free of SAT-1 viruses. Consequently, these vaccines do not provide any protection against the recently emerged SAT-1 viruses, which is what we refer to as the inadequacy of these vaccines.  We added the strains that were administered in Syria as vaccine in previous years in the discussion section(page 19). It should be noted that Syria cannot have its vaccines tracked in the same way as in stable countries. This is due to the significant chaos in the vaccine market over the past 15 years and the influx of many smuggled vaccines from Lebanon, Turkey, and Iraq of unknown origin . | 2) Insufficient description of vaccination practices  The manuscript mentions that vaccines used in Syria include serotypes O, A, and Asia-1, but provides no further detail.  Missing elements:  * vaccine type (imported/local)  * vaccine strains  * antigenic relevance to circulating viruses  This is particularly important given the discussion of vaccine failure.  Required revision  * Provide detailed information on vaccination practices  * Discuss mismatch between circulating SAT-1 and vaccine composition  * Avoid unsupported claims regarding vaccine failure |
| We fully agree with the reviewer's observation, and we acknowledge the limitations of this work and its small sample size. This is why we began the title with the word "Preliminary," and the two samples sequenced were selected based on their higher DNA concentration, as noted in the article. (page 14)  We have tried, as much as possible, to emphasize the word "hypothesis" or "assumption" and avoid generalizations.  Acknowledge limitations explicitly: done, (pages 3,23) | 3) Limited sequencing dataset  Only two isolates were sequenced despite six positive farms.  This limits:  * representativeness * phylogenetic inference * epidemiological conclusions  Required revision  * Justify sample selection * Acknowledge limitations explicitly * Avoid broad generalizations |
| We completely agree, as all points in the discussion section were preceded by the word "hypothesis" or "suggestion." The words indicating this are highlighted in yellow. | 4) Overinterpretation of phylogenetic findings  The manuscript proposes:  * transmission routes across countries * shared outbreak origins * potential vaccine-related issues  These conclusions are not sufficiently supported by:  * small sample size * partial VP1 sequencing  Required revision  * Clearly distinguish speculation from evidence * Reduce strength of causal claims |
| *nuclease-free water was used as a negative control (we added it).  *no positive was used.  * We adhered strictly and closely to the protocol described in reference No. 7 (Knowles NJ, Wadsworth J, Bachanek-Bankowska K, King DP. VP1 sequencing protocol for foot and mouth disease virus molecular epidemiology. Revue scientifique et technique (International Office of Epizootics). 2016;35(3):741-55. Epub 2016/01/01. doi: 10.20506/rst.35.3.2565. PubMed PMID: 28332654.), and therefore each sample underwent two or three PCR reactions according to the serotype and primers mentioned in the reference.  *Sequencing platform: Sanger. (we added it)  * quality control: Chromatograms were manually inspected, Ensuring the quality and accuracy of the results and the signal specific to the nucleotides in the region responsible for VP1 (we added it).  * consensus generation: Multiple PCR results were sequenced for each sample in both directions and were matched to confirm the validity of the results; no assembly was required to obtain the complete sequence.  Phylogenetics * alignment details, model justification ,reference sequence list: All the information referred to in the Materials and Methods section, and details of the reference sequences and their numbers, are mentioned and further clarified in Figure 3.  The Maximum Likelihood (ML) method was employed as it provides a robust statistical framework for phylogenetic inference. The Tamura-Nei model was selected as the best-fit substitution model according to the BIC scores calculated in MEGA11. To ensure the reliability and statistical support of the tree nodes, 1000 bootstrap replicates were performed, which is the standard threshold for high-confidence phylogenetic analysis. | 5) Methodological transparency is insufficient  Important details are missing:  RT-PCR * controls (positive/negative) * validation procedures Sequencing * platform (e.g., Sanger) * quality control * consensus generation Phylogenetics * alignment details * model justification * reference sequence list  Required revision Provide sufficient methodological detail to ensure reproducibility. |
| We completely agree.  Done | 6) Data availability statement is incorrect  The manuscript states:  “No data was generated by this study”  This is incorrect.  Required revision  Replace with a compliant statement including GenBank accession numbers. |
| Done. (page 23) | 7) Vaccine update recommendation requires stronger evidence and broader surveillance (critical point)  The manuscript recommends updating vaccination strategies to include SAT-1/III strains.  While this is a reasonable consideration, the recommendation is premature and insufficiently supported.  Key concerns:  * Conclusions are based on only two sequenced isolates * It is unclear whether: * SAT-1 Topotype III is the only circulating lineage * other SAT-1 topotypes (e.g., Topotype I) may also be present * No antigenic matching data are provided  Required revision  The authors should adopt a more evidence-based and cautious interpretation:  * Vaccine strain selection in FMD should rely on: * large-scale molecular surveillance across regions and hosts * continuous sequencing of circulating strains * antigenic matching studies (e.g., virus neutralization tests, r1-values)  The recommendation should be revised along the following lines:  “While the detection of SAT-1/III highlights a potential gap in current vaccination strategies, expanded nationwide surveillance and antigenic characterization are required to determine the diversity of SAT-1 strains circulating in Syria and to inform evidence-based vaccine strain selection This will align the manuscript with established FAO/WRLFMD principles and avoid premature policy recommendations. |

| **Reviewer #3** | **Author's answer** |
| --- | --- |
| A very recent study showed that this strain was also responsible from the outbreaks in Iran. You may add this reference before publication. | Done. (page 22) |
| The vaccines used in that country may help to understand possible source of the outbreaks. Full genome sequencing may also help to date the outbreaks. | We have added some points regarding the vaccines used in Syria over the past years. It should be noted that Syria cannot have its vaccines tracked in the same way as in stable countries. This is due to the significant chaos in the vaccine market over the past 15 years and the influx of many smuggled vaccines from Lebanon, Turkey, and Iraq of unknown origin(page 19).  We fully agree with the references regarding the importance of whole-genome sequencing, something we will try to accomplish in the coming months if the necessary financial and technical resources become available. |

The manuscript has been resubmitted to your journal. We are looking forward to your response.

Best Regards

corresponding author on behalf of all authors

Morshed Kassouha
