# Supplementary material for: Preliminary molecular characterization of foot-and-mouth disease virus SAT-1 topotype III associated with recent cattle outbreaks in Syria
Source: PLoS One. 2026 Jul 14;21(7):e0353862. doi: 10.1371/journal.pone.0353862 (PMC13367722; doi:10.1371/journal.pone.0353862)
Supplement: Attachment — Submitted filename: Response_to_Reviewers_auresp_2.docx [file pone.0353862.s003.docx]

**Response Letter**

Dear Editor-in-Chief,

We would like to thank you and the Reviewer for your useful comments and revises about our manuscript. The detailed corrections are listed below:

| **Author's answer** | **Journal Requirements** |
| --- | --- |
| Done. | 1. If the reviewer comments include a recommendation to cite specific previously published works, please review and evaluate these publications to determine whether they are relevant and should be cited. There is no requirement to cite these works unless the editor has indicated otherwise. |
| The reference list has been reviewed, minor typographical errors have been rectified, and no research was identified as having been retracted. | 2. Please review your reference list to ensure that it is complete and correct. If you have cited papers that have been retracted, please include the rationale for doing so in the manuscript text, or remove these references and replace them with relevant current references. Any changes to the reference list should be mentioned in the rebuttal letter that accompanies your revised manuscript. If you need to cite a retracted article, indicate the article’s retracted status in the References list and also include a citation and full reference for the retraction notice. |

| **Author's answer** | **Reviewer#2** |
| --- | --- |
| Done.    -page 2, line 16.  -page 14, lines 1-2.  -page 20, line 21. | 1. Statements referring to the “first detection” of SAT-1 in Syria should be revised to “first molecularly confirmed detection” or “first molecular characterization” to avoid overstatement. |
| Done.    -page 2, line 6.  -page 20, line 15. | 2. Conclusions regarding vaccine failure should be moderated, as no vaccine efficacy data were generated in the present study. The discussion should instead refer to a potential vaccine mismatch or insufficient antigenic coverage. |
| Some sentences were added and others were modified.  -page 21, line 14.  -page 21, line 21.  -page 22, lines 2-6. | 3. Some epidemiological interpretations regarding virus origin and transmission routes remain speculative and should be expressed more cautiously. |
| -page 19, lines 13-14.  -page 19, line 20. | 4. Information based on personal communications should be replaced with citable references where possible or removed. |
| Done | 5. The reference list requires careful verification. In particular, Reference 22 contains an apparent error in the first author’s initials (“Abousenna MJV”), which should be corrected to “Abousenna MS.” The authors are encouraged to check the entire bibliography for similar citation errors. |
| -page 3, line 2.  -page 11, lines 4-5.  -page 11, line 13.  -page 12, lines 7-9.  -page 14, lines 10-12.  -page 15, lines 1-3.  -page 19, lines 18-20. | 6. Minor English-language editing is still recommended to improve clarity and readability. |

The manuscript has been resubmitted to your journal. We are looking forward to your response.

Best Regards

corresponding author on behalf of all authors

Morshed Kassouha
