# Supplementary material for: Preliminary molecular characterization of foot-and-mouth disease virus SAT-1 topotype III associated with recent cattle outbreaks in Syria
Source: PLoS One. 2026 Jul 14;21(7):e0353862. doi: 10.1371/journal.pone.0353862 (PMC13367722; doi:10.1371/journal.pone.0353862)
Supplement: Peer review history

# Peer review: Decision Letter 0

**DOI:** 10.1371/journal.pone.0353862.r001

**Authors:** Mohamed Abousenna

29 Apr 2026

PONE-D-26-16745

Preliminary Molecular Characterization of Foot-and-Mouth Disease Virus SAT-1 Topotype III Associated with Recent Cattle Outbreaks in Syria

PLOS One

Dear Dr. Kassouha,

Thank you for submitting your manuscript to PLOS ONE. After careful consideration, we feel that it has merit but does not fully meet PLOS ONE’s publication criteria as it currently stands. Therefore, we invite you to submit a revised version of the manuscript that addresses the points raised during the review process.

Please submit your revised manuscript by Jun 13 2026 11:59PM. If you will need more time than this to complete your revisions, please reply to this message or contact the journal office at plosone@plos.org. When you're ready to submit your revision, log on to https://www.editorialmanager.com/pone/ and select the 'Submissions Needing Revision' folder to locate your manuscript file.

Please include the following items when submitting your revised manuscript:

- A letter that responds to each point raised by the academic editor and reviewer(s). You should upload this letter as a separate file labeled 'Response to Reviewers'.
- A marked-up copy of your manuscript that highlights changes made to the original version. You should upload this as a separate file labeled 'Revised Manuscript with Track Changes'.
- An unmarked version of your revised paper without tracked changes. You should upload this as a separate file labeled 'Manuscript'.

If you would like to make changes to your financial disclosure, please include your updated statement in your cover letter. Guidelines for resubmitting your figure files are available below the reviewer comments at the end of this letter.

If applicable, we recommend that you deposit your laboratory protocols in protocols.io to enhance the reproducibility of your results. Protocols.io assigns your protocol its own identifier (DOI) so that it can be cited independently in the future. For instructions see: https://journals.plos.org/plosone/s/submission-guidelines#loc-laboratory-protocols. Additionally, PLOS ONE offers an option for publishing peer-reviewed Lab Protocol articles, which describe protocols hosted on protocols.io. Read more information on sharing protocols at https://plos.org/protocols?utm_medium=editorial-email&utm_source=authorletters&utm_campaign=protocols.

As the corresponding author, your ORCID iD is verified in the submission system and will appear in the published article. PLOS supports the use of ORCID, and we encourage all coauthors to register for an ORCID iD and use it as well. Please encourage your coauthors to verify their ORCID iD within the submission system before final acceptance, as unverified ORCID iDs will not appear in the published article. *Only*  the individual author can complete the verification step; PLOS staff *cannot*  verify ORCID iDs on behalf of authors.

We look forward to receiving your revised manuscript.

Kind regards,

Mohamed Samy Abousenna, Ph.D

Academic Editor

PLOS One

**Journal Requirements:**

When submitting your revision, we need you to address these additional requirements.

1. Please ensure that your manuscript meets PLOS ONE's style requirements, including those for file naming. The PLOS ONE style templates can be found at

https://journals.plos.org/plosone/s/file?id=wjVg/PLOSOne_formatting_sample_main_body.pdf and

https://journals.plos.org/plosone/s/file?id=ba62/PLOSOne_formatting_sample_title_authors_affiliations.pdf

2. Please provide a complete Data Availability Statement in the submission form, ensuring you include all necessary access information or a reason for why you are unable to make your data freely accessible. If your research concerns only data provided within your submission, please write "All data are in the manuscript and/or supporting information files" as your Data Availability Statement.

3. We note that Figure 1 in your submission contain map images which may be copyrighted. All PLOS content is published under the Creative Commons Attribution License (CC BY 4.0), which means that the manuscript, images, and Supporting Information files will be freely available online, and any third party is permitted to access, download, copy, distribute, and use these materials in any way, even commercially, with proper attribution. For these reasons, we cannot publish previously copyrighted maps or satellite images created using proprietary data, such as Google software (Google Maps, Street View, and Earth). For more information, see our copyright guidelines: http://journals.plos.org/plosone/s/licenses-and-copyright.

We require you to either (1) present written permission from the copyright holder to publish these figures specifically under the CC BY 4.0 license, or (2) remove the figures from your submission:

1. You may seek permission from the original copyright holder of Figure 1 to publish the content specifically under the CC BY 4.0 license.

We recommend that you contact the original copyright holder with the Content Permission Form (http://journals.plos.org/plosone/s/file?id=7c09/content-permission-form.pdf) and the following text:

“I request permission for the open-access journal PLOS ONE to publish XXX under the Creative Commons Attribution License (CCAL) CC BY 4.0 (http://creativecommons.org/licenses/by/4.0/). Please be aware that this license allows unrestricted use and distribution, even commercially, by third parties. Please reply and provide explicit written permission to publish XXX under a CC BY license and complete the attached form.”

Please upload the completed Content Permission Form or other proof of granted permissions as an "Other" file with your submission.

In the figure caption of the copyrighted figure, please include the following text: “Reprinted from [ref] under a CC BY license, with permission from [name of publisher], original copyright [original copyright year].”

2. If you are unable to obtain permission from the original copyright holder to publish these figures under the CC BY 4.0 license or if the copyright holder’s requirements are incompatible with the CC BY 4.0 license, please either i) remove the figure or ii) supply a replacement figure that complies with the CC BY 4.0 license. Please check copyright information on all replacement figures and update the figure caption with source information. If applicable, please specify in the figure caption text when a figure is similar but not identical to the original image and is therefore for illustrative purposes only.

The following resources for replacing copyrighted map figures may be helpful:

USGS National Map Viewer (public domain): http://viewer.nationalmap.gov/viewer/

The Gateway to Astronaut Photography of Earth (public domain): http://eol.jsc.nasa.gov/sseop/clickmap/

Maps at the CIA (public domain): https://www.cia.gov/library/publications/the-world-factbook/index.html and https://www.cia.gov/library/publications/cia-maps-publications/index.html

NASA Earth Observatory (public domain): http://earthobservatory.nasa.gov/

Landsat: http://landsat.visibleearth.nasa.gov/

USGS EROS (Earth Resources Observatory and Science (EROS) Center) (public domain): http://eros.usgs.gov/#

Natural Earth (public domain): http://www.naturalearthdata.com/

4. We note that Figure 2 in your submission contain copyrighted images. All PLOS content is published under the Creative Commons Attribution License (CC BY 4.0), which means that the manuscript, images, and Supporting Information files will be freely available online, and any third party is permitted to access, download, copy, distribute, and use these materials in any way, even commercially, with proper attribution. For more information, see our copyright guidelines: http://journals.plos.org/plosone/s/licenses-and-copyright.

We require you to either (1) present written permission from the copyright holder to publish these figures specifically under the CC BY 4.0 license, or (2) remove the figures from your submission:

1. You may seek permission from the original copyright holder of Figure 2  to publish the content specifically under the CC BY 4.0 license.

We recommend that you contact the original copyright holder with the Content Permission Form (http://journals.plos.org/plosone/s/file?id=7c09/content-permission-form.pdf) and the following text:

“I request permission for the open-access journal PLOS ONE to publish XXX under the Creative Commons Attribution License (CCAL) CC BY 4.0 (http://creativecommons.org/licenses/by/4.0/). Please be aware that this license allows unrestricted use and distribution, even commercially, by third parties. Please reply and provide explicit written permission to publish XXX under a CC BY license and complete the attached form.”

Please upload the completed Content Permission Form or other proof of granted permissions as an "Other" file with your submission.

In the figure caption of the copyrighted figure, please include the following text: “Reprinted from [ref] under a CC BY license, with permission from [name of publisher], original copyright [original copyright year].”

2. If you are unable to obtain permission from the original copyright holder to publish these figures under the CC BY 4.0 license or if the copyright holder’s requirements are incompatible with the CC BY 4.0 license, please either i) remove the figure or ii) supply a replacement figure that complies with the CC BY 4.0 license. Please check copyright information on all replacement figures and update the figure caption with source information. If applicable, please specify in the figure caption text when a figure is similar but not identical to the original image and is therefore for illustrative purposes only.

5. If the reviewer comments include a recommendation to cite specific previously published works, please review and evaluate these publications to determine whether they are relevant and should be cited. There is no requirement to cite these works unless the editor has indicated otherwise.

[Note: HTML markup is below. Please do not edit.]

Reviewers' comments:

Reviewer's Responses to Questions

**Comments to the Author**

1. Is the manuscript technically sound, and do the data support the conclusions?

The manuscript must describe a technically sound piece of scientific research with data that supports the conclusions. Experiments must have been conducted rigorously, with appropriate controls, replication, and sample sizes. The conclusions must be drawn appropriately based on the data presented.

Reviewer #1: Yes

Reviewer #2: Yes

Reviewer #3: Partly

**********

2. Has the statistical analysis been performed appropriately and rigorously?

Reviewer #1: Yes

Reviewer #2: N/A

Reviewer #3: N/A

**********

3. Have the authors made all data underlying the findings in their manuscript fully available?

The PLOS Data policy requires authors to make all data underlying the findings described in their manuscript fully available without restriction, with rare exception (please refer to the Data Availability Statement in the manuscript PDF file). The data should be provided as part of the manuscript or its supporting information, or deposited to a public repository. For example, in addition to summary statistics, the data points behind means, medians and variance measures should be available. If there are restrictions on publicly sharing data—e.g. participant privacy or use of data from a third party—those must be specified.

Reviewer #1: Yes

Reviewer #2: No

Reviewer #3: Yes

**********

4. Is the manuscript presented in an intelligible fashion and written in standard English?

PLOS ONE does not copyedit accepted manuscripts, so the language in submitted articles must be clear, correct, and unambiguous. Any typographical or grammatical errors should be corrected at revision, so please note any specific errors here.

Reviewer #1: Yes

Reviewer #2: Yes

Reviewer #3: Yes

**********

5. Review Comments to the Author

Please use the space provided to explain your answers to the questions above. You may also include additional comments for the author, including concerns about dual publication, research ethics, or publication ethics. (Please upload your review as an attachment if it exceeds 20,000 characters)

Reviewer #1: 1. Data Availability Statement is incorrect)The authors state: “No data was generated by this study.” This is factually incorrect. Two novel VP1 sequences were generated and submitted to GenBank (accessions PZ124873, PZ124874). The data availability statement must be revised accordingly, e.g.:“The VP1 sequence data generated during this study are available in GenBank under accessions PZ124873 and PZ124874. Other relevant data are within the manuscript.”

2. Sample size and representativeness are very limited. Only six farms were sampled, with sequencing performed on just two isolates. Given the claim of a“complex epidemiological shift,”the authors should acknowledge this as a preliminary observation and avoid overgeneralizing to all of Syria.

3. Missing critical methodological details. PCR annealing temperatures are vaguely reported as “50, 55, or 60°C depending on serotype.”The exact temperature used for SAT-1 must be stated.

4. Phylogenetic tree presentation is poor.(1) Figure 3 is unreadable in the current format (font too small, branches unclear).(2) Bootstrap values are mentioned but not clearly shown on key nodes.(3) The tree should be simplified or split into a main figure + supplementary.

5. Unsupported claims in Discussion.(1) “Virus did not escape from vaccine production facilities”–no data support this. Speculation should be removed or clearly labeled as hypothesis.(2)“Egypt and Jordan are not the source” while plausible, absence of evidence is not evidence of absence. Tone should be more cautious.

6. Ethics statement issues. Verbal consent from farmers is noted but not in line with standard ethical guidelines for animal studies. Written consent or a formal waiver should be documented. The statement should clarify whether any animals were euthanized (apparently none).

6.Writing & Language

(1) Line 2 (page 7): “outbreak”→should be “outbreaks” (plural). Line 5 (page 7): “have raised”→“has raised” (referring to “recent outbreak”). Several sentences are overly long and complex; consider splitting for clarity.

7. Results section. The finding of 8 Syrian O/ME-SA/SA-2018 sequences (page 20) is interesting but feels disconnected from the main SAT-1 focus. This should be briefly integrated into the Discussion, not just reported.

(1)Figure 2 (clinical photos) is useful but lacks scale bars or annotations.

(2) Figure 4 (amino acid alignment) could be merged into a supplementary table.

Reviewer #2: Major Comments

1) Inadequate historical context of FMD in Syria (critical issue)

The manuscript does not provide a comprehensive epidemiological history of FMD in Syria and instead frames the findings as:

“first documented detection of SAT-1 in Syria”

This claim is insufficiently supported and potentially misleading, given that:

* FMD has been historically reported in Syria with multiple serotypes

* SAT-1 circulation has been documented historically in the region

Required revision

* Clarify whether this represents:

* first recent detection

* first molecular characterization

* or first identification of Topotype III

* Include a structured historical overview of FMD circulation in Syria

2) Insufficient description of vaccination practices

The manuscript mentions that vaccines used in Syria include serotypes O, A, and Asia-1, but provides no further detail.

Missing elements:

* vaccine type (imported/local)

* vaccine strains

* antigenic relevance to circulating viruses

This is particularly important given the discussion of vaccine failure.

Required revision

* Provide detailed information on vaccination practices

* Discuss mismatch between circulating SAT-1 and vaccine composition

* Avoid unsupported claims regarding vaccine failure

3) Limited sequencing dataset

Only two isolates were sequenced despite six positive farms.

This limits:

* representativeness

* phylogenetic inference

* epidemiological conclusions

Required revision

* Justify sample selection

* Acknowledge limitations explicitly

* Avoid broad generalizations

4) Overinterpretation of phylogenetic findings

The manuscript proposes:

* transmission routes across countries

* shared outbreak origins

* potential vaccine-related issues

These conclusions are not sufficiently supported by:

* small sample size

* partial VP1 sequencing

Required revision

* Clearly distinguish speculation from evidence

* Reduce strength of causal claims

5) Methodological transparency is insufficient

Important details are missing:

RT-PCR

* controls (positive/negative)

* validation procedures

Sequencing

* platform (e.g., Sanger)

* quality control

* consensus generation

Phylogenetics

* alignment details

* model justification

* reference sequence list

Required revision

Provide sufficient methodological detail to ensure reproducibility.

6) Data availability statement is incorrect

The manuscript states:

“No data was generated by this study”

This is incorrect.

Required revision

Replace with a compliant statement including GenBank accession numbers.

7) Vaccine update recommendation requires stronger evidence and broader surveillance (critical point)

The manuscript recommends updating vaccination strategies to include SAT-1/III strains.

While this is a reasonable consideration, the recommendation is premature and insufficiently supported.

Key concerns:

* Conclusions are based on only two sequenced isolates

* It is unclear whether:

* SAT-1 Topotype III is the only circulating lineage

* other SAT-1 topotypes (e.g., Topotype I) may also be present

* No antigenic matching data are provided

Required revision

The authors should adopt a more evidence-based and cautious interpretation:

* Vaccine strain selection in FMD should rely on:

* large-scale molecular surveillance across regions and hosts

* continuous sequencing of circulating strains

* antigenic matching studies (e.g., virus neutralization tests, r1-values)

The recommendation should be revised along the following lines:

“While the detection of SAT-1/III highlights a potential gap in current vaccination strategies, expanded nationwide surveillance and antigenic characterization are required to determine the diversity of SAT-1 strains circulating in Syria and to inform evidence-based vaccine strain selection.” According to Abousenna, M.S., Khafagy, H.A., Mohamed, A.A. et al. Emergency response for recently

isolated Foot and Mouth Disease virus type A Africa in Egypt 2022. Scientific Reports , 15, 4475

(2025). https://doi.org/10.1038/s41598-025-88906-4

Abousenna M.S. Emergency evaluation for existing vaccine against recently isolated Foot and

Mouth Disease Virus type SAT2 in Egypt 2018. VacciMonitor , 31(1):9-14 (2022).

This will align the manuscript with established FAO/WRLFMD principles and avoid premature policy recommendations.

Reviewer #3: Dear Authors;

The study contains extremely important findings. Firstly, This is one of the few reports from Syria for a long time. The second but not the least important finding is the circulating virus which is very closely related to a vaccine strain. A very recent study showed that this strain was also responsible from the outbreaks in Iran. You may add this reference before publication. This study had some flaws such as very limited number of samples and lack of serological data that might help to understand the complete picture. Also, vaccines used in that country may help to understand possible source of the outbreaks. Full genome sequencing may also help to date the outbreaks.

**********

6. PLOS authors have the option to publish the peer review history of their article (what does this mean?). If published, this will include your full peer review and any attached files.

If you choose “no”, your identity will remain anonymous but your review may still be made public.

**Do you want your identity to be public for this peer review?**  For information about this choice, including consent withdrawal, please see our Privacy Policy.

Reviewer #1: No

Reviewer #2: No

Reviewer #3: No

**********

[NOTE: If reviewer comments were submitted as an attachment file, they will be attached to this email and accessible via the submission site. Please log into your account, locate the manuscript record, and check for the action link "View Attachments". If this link does not appear, there are no attachment files.]

To ensure your figures meet our technical requirements, please review our figure guidelines: https://journals.plos.org/plosone/s/figures

You may also use PLOS’s free figure tool, NAAS, to help you prepare publication quality figures: https://journals.plos.org/plosone/s/figures#loc-tools-for-figure-preparation.

NAAS will assess whether your figures meet our technical requirements by comparing each figure against our figure specifications.

# Author comment: Author response to Decision Letter 1

**DOI:** 10.1371/journal.pone.0353862.r002

22 May 2026

Dear Editor-in-Chief,

We would like to thank you and the Reviewers for your useful comments and revises about our manuscript.

The authors are open to any further modifications and look forward to your expert opinion.

Sincerely,

Morshed

--------------------------------------------------------------------------------------------

Journal Requirements:

1.Please ensure that your manuscript meets PLOS ONE's style requirements, including those for file naming.

Author's answer:

Done.

2. Please provide a complete Data Availability Statement in the submission form

Author's answer:

Done.

3. We note that Figure 1 in your submission contain map images which may be copyrighted.

Author's answer:

Done, we replaced the figure 1 with one from another website (Syria - CIA), and according to the source, we do not need their permission to use or modify it.

4. We note that Figure 2 in your submission contain copyrighted images.

Author's answer:

Figure 2 is composed of photographs captured by the authors, which have not been published previously, illustrating instances of foot-and-mouth disease from the farms where the samples were collected. We relinquish all rights to their usage to the journal and authorize their use by a third party or republication as considered suitable.

------------------------------------------------------------------------------------------------------------------------------

Reviewer #1

1- Data Availability Statement is incorrect, the authors state: “No data was generated by this study.” This is factually incorrect. Two novel VP1 sequences were generated and submitted to GenBank (accessions PZ124873, PZ124874). The data availability statement must be revised accordingly, e.g.:“The VP1 sequence data generated during this study are available in GenBank under accessions PZ124873 and PZ124874. Other relevant data are within the manuscript.”

Author's answer:

We completely agree.

Done.

2. Sample size and representativeness are very limited. Only six farms were sampled, with sequencing performed on just two isolates. Given the claim of a“complex epidemiological shift,”the authors should acknowledge this as a preliminary observation and avoid overgeneralizing to all of Syria.

Author's answer:

Certainly, the number of samples is small and limited, and we fully agree with the reviewer's observation, which is why we wrote the word "preliminary" at the beginning of the title. On the other hand, the outbreaks that occurred in Syria during 2025 and the current year were extremely severe and their spread was terrifying, with most of the previously used vaccines proving ineffective. If we take this into account, along with the large spread that accompanied the outbreaks that occurred in Lebanon, the country neighboring Syria, which were also caused by FMDV SAT-1/III, this aligns with the result of this work, which definitively confirmed its presence in Syria for the first time. Furthermore, the presence of a new strain of the virus, serotype O(O/ME-SA/SA-2018 lineage), introduced into Pool-3 from Pool-2, as we mentioned in the discussion, was also recorded. All of the above points suggest a complex epidemiological situation that requires close monitoring, especially given the authorities' inability to control borders and animal movement, and the indiscriminate use of vaccines from various sources without any controls.

3. Missing critical methodological details. PCR annealing temperatures are vaguely reported as “50, 55, or 60°C depending on serotype.”The exact temperature used for SAT-1 must be stated.

Author's answer:

Done. (page 11)

4. Phylogenetic tree presentation is poor.(1) Figure 3 is unreadable in the current format (font too small, branches unclear).(2) Bootstrap values are mentioned but not clearly shown on key nodes.(3) The tree should be simplified or split into a main figure + supplementary.

Author's answer:

The figure has been modified to become clearer, and the reviewer can download the figure in full resolution from the link at the top of the page. Thank you for your note.

5. Unsupported claims in Discussion.(1) “Virus did not escape from vaccine production facilities”–no data support this. Speculation should be removed or clearly labeled as hypothesis.(2)“Egypt and Jordan are not the source” while plausible, absence of evidence is not evidence of absence. Tone should be more cautious.

Author's answer:

Claim (1): We fully agree with the reviewer's statement, and it was emphasized that this is a hypothesis (page 21, line 4). It should be noted that what was mentioned in the discussion chapter was taken literally from the referenced WOAH/FAO/WRLFMD report, as well as from a recently published article that highlighted the significant similarity between the SAT-1/III foot-and-mouth disease virus, which caused large outbreaks in Iran, and the BOT/1/77 reference strain used in vaccine production. However, this claim certainly remains a hypothesis.

Claim (2): That's a correct point. As we hypothesized in the discussion section regarding this issue, we based our assumption on the evidence available in published scientific articles and the sequences deposited in the GenBank database or the International Reference Laboratory for Foot-and-Mouth Disease. It's worth noting that the veterinary authorities in Egypt and Jordan are among the best in the region, and both countries have universities with advanced laboratories capable of detecting the viruses causing outbreaks.

6. Ethics statement issues. Verbal consent from farmers is noted but not in line with standard ethical guidelines for animal studies. Written consent or a formal waiver should be documented. The statement should clarify whether any animals were euthanized (apparently none).

Author's answer:

Obtaining written consent is certainly preferable (especially for studies conducted on humans, regardless of their form).

In this study, it was impossible to obtain written consent from some farmers whose animals were sampled because they are illiterate. We explained to them that these samples collected from their animals would contribute to identifying the pathogen affecting their livestock, and that the results would be used and published in scientific research to enhance our understanding of this virus. They were happy to agree.

A statement was added indicating that no animals were euthanized in this study. (page 9, line 2)

7.Writing & Language

(1) Line 2 (page 7): “outbreak”→should be “outbreaks” (plural). Line 5 (page 7): “have raised”→“has raised” (referring to “recent outbreak”). Several sentences are overly long and complex; consider splitting for clarity.

Author's answer:

Done.

8. Results section. The finding of 8 Syrian O/ME-SA/SA-2018 sequences (page 20) is interesting but feels disconnected from the main SAT-1 focus. This should be briefly integrated into the Discussion, not just reported.

Author's answer:

This point was reported at the end of the results, and the significance of the O/ME-SA/SA-2018 lineage is discussed in several places in the discussion section (page 22).

9. (1)Figure 2 (clinical photos) is useful but lacks scale bars or annotations.

Author's answer:

The figure's annotations are detailed in the caption on page 9. However, given that the images depict clinical symptoms—some closely focused on the mouth to illustrate the lesions, while others are taken from a distance to capture the animal's full head and excessive salivation—it seems quite challenging to define a single scale bar that accurately represents the entire figure.

10.(2) Figure 4 (amino acid alignment) could be merged into a supplementary table.

Author's answer:

We appreciate the reviewer's observation. While we are not rigidly attached to our viewpoint and are receptive to modifications, we propose keeping the figure with an added explanatory sentence in the caption. Figure 4 illustrates the variations in amino acids and their respective positions among the Syrian sequences, as well as in comparison to the significant reference strain BOT/1/77.

The reader can readily identify the two points of divergence where the green line is interrupted. The first point of divergence occurs between the sequence of the virus studied from Homs Governorate, Syria, and the other two sequences. The second point highlights the differences between the reference strain BOT/1/77 and the Syrian sequences. The green line aids in simplifying and enhancing the visualization of these differences, and we have included a sentence in the caption to clarify this.

---------------------------------------------------------------------------------------------------------------------------------------------------------------

Reviewer#2

1) Inadequate historical context of FMD in Syria (critical issue)

The manuscript does not provide a comprehensive epidemiological history of FMD in Syria and instead frames the findings as:

“first documented detection of SAT-1 in Syria”

This claim is insufficiently supported and potentially misleading, given that:

* FMD has been historically reported in Syria with multiple serotypes

* SAT-1 circulation has been documented historically in the region

Required revision

* Clarify whether this represents:

* first recent detection

* first molecular characterization

* or first identification of Topotype III

* Include a structured historical overview of FMD circulation in Syria

Author's answer:

No research on FMD virus for molecular characterization has been undertaken by Syrian scholars within Syria over the last forty years. All existing data regarding serotypes and the molecular characterization of the foot-and-mouth disease virus has been derived from samples collected by researchers outside of Syria, which were molecularly characterized at the WRLFMD laboratory during the 1960s, 1980s, 1990s, and in 2002. Furthermore, the sequences referenced in our article were molecularly characterized in Turkey in 2025 and subsequently deposited in GenBank at the start of 2026 (it is important to note that all serotypes mentioned were of types O and A). This situation arose because the state historically prohibited any research on foot-and-mouth disease, whether serological or molecular, due to its repercussions on the economy and animal exports. However, since 2025, such research has become feasible. Consequently, this study aligns precisely with the reviewer's comment, as it represents the first identification of the causative agent SAT-1 topotype III and its initial molecular characterization.

We included a concise historical overview in the introduction regarding the limited knowledge about Syria, and we will address all the requests made by the reviewer. (pages 2,6,21)

2) Insufficient description of vaccination practices

The manuscript mentions that vaccines used in Syria include serotypes O, A, and Asia-1, but provides no further detail.

Missing elements:

* vaccine type (imported/local)

* vaccine strains

* antigenic relevance to circulating viruses

This is particularly important given the discussion of vaccine failure.

Required revision

* Provide detailed information on vaccination practices

* Discuss mismatch between circulating SAT-1 and vaccine composition

* Avoid unsupported claims regarding vaccine failure

Author's answer:

All vaccines that have been officially administered in Syria over the last twenty years, up to 2025, were imported from a singular supplier and were entirely free of SAT-1 viruses. Consequently, these vaccines do not provide any protection against the recently emerged SAT-1 viruses, which is what we refer to as the inadequacy of these vaccines.

We added the strains that were administered in Syria as vaccine in previous years in the discussion section(page 19). It should be noted that Syria cannot have its vaccines tracked in the same way as in stable countries. This is due to the significant chaos in the vaccine market over the past 15 years and the influx of many smuggled vaccines from Lebanon, Turkey, and Iraq of unknown origin .

3) Limited sequencing dataset

Only two isolates were sequenced despite six positive farms.

This limits:

* representativeness

* phylogenetic inference

* epidemiological conclusions

Required revision

* Justify sample selection

* Acknowledge limitations explicitly

* Avoid broad generalizations

Author's answer

We fully agree with the reviewer's observation, and we acknowledge the limitations of this work and its small sample size. This is why we began the title with the word "Preliminary," and the two samples sequenced were selected based on their higher DNA concentration, as noted in the article. (page 14)

We have tried, as much as possible, to emphasize the word "hypothesis" or "assumption" and avoid generalizations.

Acknowledge limitations explicitly: done, (pages 3,23)

4) Overinterpretation of phylogenetic findings

The manuscript proposes:

* transmission routes across countries

* shared outbreak origins

* potential vaccine-related issues

These conclusions are not sufficiently supported by:

* small sample size

* partial VP1 sequencing

Required revision

* Clearly distinguish speculation from evidence

* Reduce strength of causal claims

Author's answer:

We completely agree, as all points in the discussion section were preceded by the word "hypothesis" or "suggestion." The words indicating this are highlighted in yellow.

5) Methodological transparency is insufficient

Important details are missing:

RT-PCR

* controls (positive/negative)

* validation procedures

Sequencing

* platform (e.g., Sanger)

* quality control

* consensus generation

Phylogenetics

* alignment details

* model justification

* reference sequence list

Required revision

Provide sufficient methodological detail to ensure reproducibility.

Author's answer:

*nuclease-free water was used as a negative control (we added it).

*no positive was used.

* We adhered strictly and closely to the protocol described in reference No. 7 (Knowles NJ, Wadsworth J, Bachanek-Bankowska K, King DP. VP1 sequencing protocol for foot and mouth disease virus molecular epidemiology. Revue scientifique et technique (International Office of Epizootics). 2016;35(3):741-55. Epub 2016/01/01. doi: 10.20506/rst.35.3.2565. PubMed PMID: 28332654.), and therefore each sample underwent two or three PCR reactions according to the serotype and primers mentioned in the reference.

*Sequencing platform: Sanger. (we added it)

* quality control: Chromatograms were manually inspected, Ensuring the quality and accuracy of the results and the signal specific to the nucleotides in the region responsible for VP1 (we added it).

* consensus generation: Multiple PCR results were sequenced for each sample in both directions and were matched to confirm the validity of the results; no assembly was required to obtain the complete sequence.

Phylogenetics

* alignment details, model justification

,reference sequence list: All the information referred to in the Materials and Methods section, and details of the reference sequences and their numbers, are mentioned and further clarified in Figure 3.

The Maximum Likelihood (ML) method was employed as it provides a robust statistical framework for phylogenetic inference. The Tamura-Nei model was selected as the best-fit substitution model according to the BIC scores calculated in MEGA11. To ensure the reliability and statistical support of the tree nodes, 1000 bootstrap replicates were performed, which is the standard threshold for high-confidence phylogenetic analysis.

6) Data availability statement is incorrect

The manuscript states:

“No data was generated by this study”

This is incorrect.

Required revision

Replace with a compliant statement including GenBank accession numbers.

Author's answer

We completely agree.

Done.

7) Vaccine update recommendation requires stronger evidence and broader surveillance (critical point)

The manuscript recommends updating vaccination strategies to include SAT-1/III strains.

While this is a reasonable consideration, the recommendation is premature and insufficiently supported.

Key concerns:

* Conclusions are based on only two sequenced isolates

* It is unclear whether:

* SAT-1 Topot

Attachment Submitted filename: Response to Reviewers.docx

# Peer review: Decision Letter 1

**DOI:** 10.1371/journal.pone.0353862.r003

**Authors:** Mohamed Abousenna, Mohamed Abousenna

12 Jun 2026

PONE-D-26-16745R1

Preliminary Molecular Characterization of Foot-and-Mouth Disease Virus SAT-1 Topotype III Associated with Recent Cattle Outbreaks in Syria

PLOS One

Dear Dr. Kassouha,

Thank you for submitting your manuscript to PLOS ONE. After careful consideration, we feel that it has merit but does not fully meet PLOS ONE’s publication criteria as it currently stands. Therefore, we invite you to submit a revised version of the manuscript that addresses the points raised during the review process.

Please submit your revised manuscript by Jul 27 2026 11:59PM. If you will need more time than this to complete your revisions, please reply to this message or contact the journal office at plosone@plos.org. When you're ready to submit your revision, log on to https://www.editorialmanager.com/pone/ and select the 'Submissions Needing Revision' folder to locate your manuscript file.

Please include the following items when submitting your revised manuscript:

- A letter that responds to each point raised by the academic editor and reviewer(s). You should upload this letter as a separate file labeled 'Response to Reviewers'.
- A marked-up copy of your manuscript that highlights changes made to the original version. You should upload this as a separate file labeled 'Revised Manuscript with Track Changes'.
- An unmarked version of your revised paper without tracked changes. You should upload this as a separate file labeled 'Manuscript'.

If you would like to make changes to your financial disclosure, please include your updated statement in your cover letter. Guidelines for resubmitting your figure files are available below the reviewer comments at the end of this letter.

If applicable, we recommend that you deposit your laboratory protocols in protocols.io to enhance the reproducibility of your results. Protocols.io assigns your protocol its own identifier (DOI) so that it can be cited independently in the future. For instructions see: https://journals.plos.org/plosone/s/submission-guidelines#loc-laboratory-protocols. Additionally, PLOS ONE offers an option for publishing peer-reviewed Lab Protocol articles, which describe protocols hosted on protocols.io. Read more information on sharing protocols at https://plos.org/protocols?utm_medium=editorial-email&utm_source=authorletters&utm_campaign=protocols.

As the corresponding author, your ORCID iD is verified in the submission system and will appear in the published article. PLOS supports the use of ORCID, and we encourage all coauthors to register for an ORCID iD and use it as well. Please encourage your coauthors to verify their ORCID iD within the submission system before final acceptance, as unverified ORCID iDs will not appear in the published article. *Only*  the individual author can complete the verification step; PLOS staff *cannot*  verify ORCID iDs on behalf of authors.

We look forward to receiving your revised manuscript.

Kind regards,

Mohamed Samy Abousenna, Ph.D

Academic Editor

PLOS One

Journal Requirements:

1. If the reviewer comments include a recommendation to cite specific previously published works, please review and evaluate these publications to determine whether they are relevant and should be cited. There is no requirement to cite these works unless the editor has indicated otherwise.

2. Please review your reference list to ensure that it is complete and correct. If you have cited papers that have been retracted, please include the rationale for doing so in the manuscript text, or remove these references and replace them with relevant current references. Any changes to the reference list should be mentioned in the rebuttal letter that accompanies your revised manuscript. If you need to cite a retracted article, indicate the article’s retracted status in the References list and also include a citation and full reference for the retraction notice.

[Note: HTML markup is below. Please do not edit.]

Reviewer's Responses to Questions

**Comments to the Author**

1. If the authors have adequately addressed your comments raised in a previous round of review and you feel that this manuscript is now acceptable for publication, you may indicate that here to bypass the “Comments to the Author” section, enter your conflict of interest statement in the “Confidential to Editor” section, and submit your "Accept" recommendation.

Reviewer #2: All comments have been addressed

**********

2. Is the manuscript technically sound, and do the data support the conclusions?

The manuscript must describe a technically sound piece of scientific research with data that supports the conclusions. Experiments must have been conducted rigorously, with appropriate controls, replication, and sample sizes. The conclusions must be drawn appropriately based on the data presented.

Reviewer #2: Yes

**********

3. Has the statistical analysis been performed appropriately and rigorously?

Reviewer #2: Yes

**********

4. Have the authors made all data underlying the findings in their manuscript fully available?

The PLOS Data policy requires authors to make all data underlying the findings described in their manuscript fully available without restriction, with rare exception (please refer to the Data Availability Statement in the manuscript PDF file). The data should be provided as part of the manuscript or its supporting information, or deposited to a public repository. For example, in addition to summary statistics, the data points behind means, medians and variance measures should be available. If there are restrictions on publicly sharing data—e.g. participant privacy or use of data from a third party—those must be specified.

Reviewer #2: Yes

**********

5. Is the manuscript presented in an intelligible fashion and written in standard English?

PLOS ONE does not copyedit accepted manuscripts, so the language in submitted articles must be clear, correct, and unambiguous. Any typographical or grammatical errors should be corrected at revision, so please note any specific errors here.

Reviewer #2: Yes

**********

6. Review Comments to the Author

Please use the space provided to explain your answers to the questions above. You may also include additional comments for the author, including concerns about dual publication, research ethics, or publication ethics. (Please upload your review as an attachment if it exceeds 20,000 characters)

Reviewer #2: The authors have made substantial efforts to address the reviewers’ comments, and the manuscript has improved considerably compared with the original submission. The revisions have strengthened the methodological description, clarified the molecular analyses, improved the discussion, and added important information regarding sequencing procedures, study limitations, and data availability.

The study provides valuable information on the molecular characterization of FMDV SAT-1 Topotype III detected in Syria and represents an important contribution to regional Foot-and-Mouth Disease surveillance and epidemiology.

Most of the previous comments have been adequately addressed. However, a few minor issues should be corrected before acceptance:

1. Statements referring to the “first detection” of SAT-1 in Syria should be revised to “first molecularly confirmed detection” or “first molecular characterization” to avoid overstatement.

2. Conclusions regarding vaccine failure should be moderated, as no vaccine efficacy data were generated in the present study. The discussion should instead refer to a potential vaccine mismatch or insufficient antigenic coverage.

3. Some epidemiological interpretations regarding virus origin and transmission routes remain speculative and should be expressed more cautiously.

4. Information based on personal communications should be replaced with citable references where possible or removed.

5. The reference list requires careful verification. In particular, Reference 22 contains an apparent error in the first author’s initials (“Abousenna MJV”), which should be corrected to “Abousenna MS.” The authors are encouraged to check the entire bibliography for similar citation errors.

6. Minor English-language editing is still recommended to improve clarity and readability.

Overall, the manuscript is scientifically sound, addresses an important topic, and is close to being suitable for publication. I recommend acceptance after minor revision.

**********

7. PLOS authors have the option to publish the peer review history of their article (what does this mean?). If published, this will include your full peer review and any attached files.

If you choose “no”, your identity will remain anonymous but your review may still be made public.

**Do you want your identity to be public for this peer review?**  For information about this choice, including consent withdrawal, please see our Privacy Policy.

Reviewer #2: No

**********

[NOTE: If reviewer comments were submitted as an attachment file, they will be attached to this email and accessible via the submission site. Please log into your account, locate the manuscript record, and check for the action link "View Attachments". If this link does not appear, there are no attachment files.]

To ensure your figures meet our technical requirements, please review our figure guidelines: https://journals.plos.org/plosone/s/figures

You may also use PLOS’s free figure tool, NAAS, to help you prepare publication quality figures: https://journals.plos.org/plosone/s/figures#loc-tools-for-figure-preparation.

NAAS will assess whether your figures meet our technical requirements by comparing each figure against our figure specifications.

# Author comment: Author response to Decision Letter 2

**DOI:** 10.1371/journal.pone.0353862.r004

28 Jun 2026

Reviewer#2

1. Statements referring to the “first detection” of SAT-1 in Syria should be revised to “first molecularly confirmed detection” or “first molecular characterization” to avoid overstatement.

Author's answer:

Done.

-page 2, line 16.

-page 14, lines 1-2.

-page 20, line 21.

2. Conclusions regarding vaccine failure should be moderated, as no vaccine efficacy data were generated in the present study. The discussion should instead refer to a potential vaccine mismatch or insufficient antigenic coverage.

Author's answer:

Done.

-page 2, line 6.

-page 20, line 15.

3. Some epidemiological interpretations regarding virus origin and transmission routes remain speculative and should be expressed more cautiously.

Author's Answer:

Some sentences were added and others were modified.

-page 21, line 14.

-page 21, line 21.

-page 22, lines 2-6.

4. Information based on personal communications should be replaced with citable references where possible or removed.

Author's Answer: done.

-page 19, lines 13-14.

-page 19, line 20.

5. The reference list requires careful verification. In particular, Reference 22 contains an apparent error in the first author’s initials (“Abousenna MJV”), which should be corrected to “Abousenna MS.” The authors are encouraged to check the entire bibliography for similar citation errors.

Author's Answer: done.

6. Minor English-language editing is still recommended to improve clarity and readability.

Author's Answer:

We read the manuscript, made some sentences clearer, and edited them to make them easier to read and understand, we also corrected some grammatical errors that we found.

-page 3, line 2.

-page 11, lines 4-5.

-page 11, line 13.

-page 12, lines 7-9.

-page 14, lines 10-12.

-page 15, lines 1-3.

-page 19, lines 18-20.

Attachment Submitted filename: Response_to_Reviewers_auresp_2.docx

# Peer review: Decision Letter 2

**DOI:** 10.1371/journal.pone.0353862.r005

**Authors:** Mohamed Abousenna, Mohamed Abousenna, Mohamed Abousenna

1 Jul 2026

Preliminary Molecular Characterization of Foot-and-Mouth Disease Virus SAT-1 Topotype III Associated with Recent Cattle Outbreaks in Syria

PONE-D-26-16745R2

Dear Dr. Kassouha,

We’re pleased to inform you that your manuscript has been judged scientifically suitable for publication and will be formally accepted for publication once it meets all outstanding technical requirements.

Within one week, you’ll receive an e-mail detailing the required amendments. When these have been addressed, you’ll receive a formal acceptance letter and your manuscript will be scheduled for publication.

An invoice will be generated when your article is formally accepted. Please note, if your institution has a publishing partnership with PLOS and your article meets the relevant criteria, all or part of your publication costs will be covered. Please make sure your user information is up-to-date by logging into Editorial Manager at Editorial Manager® and clicking the ‘Update My Information' link at the top of the page. For questions related to billing, please contact billing support.

If your institution or institutions have a press office, please notify them about your upcoming paper to help maximize its impact. If they’ll be preparing press materials, please inform our press team as soon as possible -- no later than 48 hours after receiving the formal acceptance. Your manuscript will remain under strict press embargo until 2 pm Eastern Time on the date of publication. For more information, please contact onepress@plos.org.

Kind regards,

Mohamed Samy Abousenna, Ph.D

Academic Editor

PLOS One

Additional Editor Comments (optional):

The reviewer comments have been adequately addressed; however, please perform a final careful editorial revision to improve the English language, grammar, consistency, and clarity throughout the manuscript before acceptance.

Reviewers' comments:

Reviewer's Responses to Questions

**Comments to the Author**

1. If the authors have adequately addressed your comments raised in a previous round of review and you feel that this manuscript is now acceptable for publication, you may indicate that here to bypass the “Comments to the Author” section, enter your conflict of interest statement in the “Confidential to Editor” section, and submit your "Accept" recommendation.

Reviewer #2: All comments have been addressed

**********

2. Is the manuscript technically sound, and do the data support the conclusions?

The manuscript must describe a technically sound piece of scientific research with data that supports the conclusions. Experiments must have been conducted rigorously, with appropriate controls, replication, and sample sizes. The conclusions must be drawn appropriately based on the data presented.

Reviewer #2: Yes

**********

3. Has the statistical analysis been performed appropriately and rigorously?

Reviewer #2: N/A

**********

4. Have the authors made all data underlying the findings in their manuscript fully available?

The PLOS Data policy requires authors to make all data underlying the findings described in their manuscript fully available without restriction, with rare exception (please refer to the Data Availability Statement in the manuscript PDF file). The data should be provided as part of the manuscript or its supporting information, or deposited to a public repository. For example, in addition to summary statistics, the data points behind means, medians and variance measures should be available. If there are restrictions on publicly sharing data—e.g. participant privacy or use of data from a third party—those must be specified.

Reviewer #2: Yes

**********

5. Is the manuscript presented in an intelligible fashion and written in standard English?

PLOS ONE does not copyedit accepted manuscripts, so the language in submitted articles must be clear, correct, and unambiguous. Any typographical or grammatical errors should be corrected at revision, so please note any specific errors here.

Reviewer #2: No

**********

6. Review Comments to the Author

Please use the space provided to explain your answers to the questions above. You may also include additional comments for the author, including concerns about dual publication, research ethics, or publication ethics. (Please upload your review as an attachment if it exceeds 20,000 characters)

Reviewer #2: (No Response)

**********

7. PLOS authors have the option to publish the peer review history of their article (what does this mean?). If published, this will include your full peer review and any attached files.

If you choose “no”, your identity will remain anonymous but your review may still be made public.

**Do you want your identity to be public for this peer review?**  For information about this choice, including consent withdrawal, please see our Privacy Policy.

Reviewer #2: No

**********

# Editor report: Acceptance letter

**DOI:** 10.1371/journal.pone.0353862.r006

**Authors:** Mohamed Abousenna, Mohamed Abousenna, Mohamed Abousenna

PONE-D-26-16745R2

PLOS One

Dear Dr. Kassouha,

I'm pleased to inform you that your manuscript has been deemed suitable for publication in PLOS One. Congratulations! Your manuscript is now being handed over to our production team.

At this stage, our production department will prepare your paper for publication. This includes ensuring the following:

* All references, tables, and figures are properly cited

* All relevant supporting information is included in the manuscript submission,

* There are no issues that prevent the paper from being properly typeset

You will receive further instructions from the production team, including instructions on how to review your proof when it is ready. Please keep in mind that we are working through a large volume of accepted articles, so please give us a few days to review your paper and let you know the next and final steps.

Lastly, if your institution or institutions have a press office, please let them know about your upcoming paper now to help maximize its impact. If they'll be preparing press materials, please inform our press team within the next 48 hours. Your manuscript will remain under strict press embargo until 2 pm Eastern Time on the date of publication. For more information, please contact onepress@plos.org.

You will receive an invoice from PLOS for your publication fee after your manuscript has reached the completed accept phase. If you receive an email requesting payment before acceptance or for any other service, this may be a phishing scheme. Learn how to identify phishing emails and protect your accounts at https://explore.plos.org/phishing.

If we can help with anything else, please email us at customercare@plos.org.

Thank you for submitting your work to PLOS ONE and supporting open access.

Kind regards,

PLOS ONE Editorial Office Staff

on behalf of

Dr. Mohamed Samy Abousenna

Academic Editor

PLOS One
